# Ginsenoside Rg1 Reduces Oxidative Stress Via Nrf2 Activation to Regulate Age-Related Mesenchymal Stem Cells Fate Switch Between Osteoblasts and Adipocytes

**DOI:** 10.1155/2022/1411354

**Published:** 2022-10-11

**Authors:** Jiying Hou, Lu Wang, Chen Wang, Ruoxiang Ma, Ziling Wang, Hanxianzhi Xiao, Di Zeng, Li Ling, Yaping Wang

**Affiliations:** ^1^Department of Histology and Embryology, Laboratory of Stem Cells and Tissue Engineering, Chongqing Medical University, Chongqing 400016, China; ^2^Chongqing Medical and Pharmaceutical College, Chongqing 401331, China; ^3^Department of Obstetrics and Gynecology, The Second Affiliated Hospital of Chongqing Medical University, Chongqing 400010, China

## Abstract

**Background:**

An important feature of aging cells is the gradual loss of physiological integrity. As aging progresses, MSCs change preferring to differentiate toward adipocytes rather than osteoblasts. Oxidative stress accumulation is an important factor in age-related bone loss. Many experiments have demonstrated the good therapeutic effect of Ginsenoside (Rg1) on oxidative stress injury. In this study, we investigated the effect of Rg1 on the osteogenic-adipogenic differentiation balance of bone marrow mesenchymal stem cells (BMMSC).

**Objective:**

To analyze the potential application value of Rg1 in the treatment of senile osteoporosis.

**Methods:**

BMMSCs were isolated from healthy donors of different ages and identified based on isotype and by multi-differentiation induction. Rg1 was used to treat BMMSCs, The differentiation propensity was analyzed by induction of differentiation assay. Antioxidant capacity of BMMSCs as measured by oxidative stress product assay Related mechanism studies were confirmed by quantitative real-time reverse transcription-polymerase chain reaction (qRT‐PCR), immunofluorescence, western blotting, and inhibitor treatment. Moreover, Observation of the effects of Rg1 on aging BMMSCs under *in vivo* conditions by treatment of aged mice with Rg1 injections.

**Results:**

Rg1 treatment rescued age‐induced switch of BMMSCs differentiation fate in vitro. In elderly people, Rg1 markedly increased osteogenic differentiation of BMMSCs by decreasing oxidative stress, while inhibiting adipogenic differentiation. However, this effect was abolished in BMMSCs by an Nrf2-inhibitor. Notably, aging mice showed a reduction in adipocyte distribution in the bone marrow and a decrease in oxidative stress products after a 3-month period of Rg1 treatment.

**Conclusion:**

We have uncovered a novel function for Rg1 that involves attenuating bone loss via Nrf2 antioxidant signaling, which in turn may potentially be utilized as a therapeutic agent for improving osteogenic differentiation in aging BMMSCs.

## 1. Introduction

Oxidative stress plays a crucial role in stem cell aging. In BMMSCs, osteogenic differentiation and stemness decrease with the accumulation of age-related oxidative stress. The lineage commitment of mesenchymal stem cells (MSCs) plays an important role in BMMSC function. As aging progresses, the differentiation tendency of MSCs changes, preferring to differentiate towards adipocytes rather than osteoblasts. An important feature of aging cells is the gradual loss of physiological integrity. In aging, cell damage accumulates over time with increased reactive oxygen species (ROS) levels to maintain cell survival. However, as the compensatory capacity reaches its limit, ROS exceeds the initial homeostatic goal and ultimately aggravates age-related damage. In bone aging, ROS disrupts bone formation by promoting the adipogenic differentiation of BMMSCs while weakening osteogenic differentiation [1].

Nuclear factor erythroid 2-related factor 2 (Nrf2) is the main regulator for maintaining cell redox homeostasis [2]. On exposure to stimuli, Nrf2 dissociates from the Kelch Like ECH Associated Protein 1 (Keap1) protein for phosphorylation to enter the nucleus. Subsequently, translational expression of downstream antioxidant enzymes is initiated in the nucleus [3]. In oxidative stress-mediated cell fate homeostasis, Nrf2 and its regulated antioxidant enzymes play an important role. It can affect the balance between osteogenesis and adipogenesis [4] and reduce mitochondrial damage [5], which in turn leads to an imbalance between bone and fat in age-related osteoporosis.

The ginsenoside Rg1 is an important antioxidant ingredient in ginseng. In the past researches, it has been reported that Rg1 can affect osteogenic differentiation in a variety of cells [6–8]. In our previous experiments, we treated human dental pulp stem cells (hDPSCs) with Rg1. The results revealed that Rg1 treatment upregulated the transcription of osteogenic genes and significantly inhibit the expression of components of the P53 pathway. These experiments provide some experimental basis for the therapeutic effect of Rg1 in promoting osteogenesis [9]. In another previous research, we found that Rg1 can alleviate the senescence of BMMSCs. At the same time, the decrease in differentiation caused by senescence is also alleviated [10]. However, the specific mechanism has not been clarified. Later, in research on senescent mesenchymal stem cells in mice, we found that Nrf2 is an important part of Rg1's resistance to the senescence of mesenchymal stem cells [11]. It is worth noting that senescence is only one important influencing factor in the aging process. There are still many other factors involved in the actual occurrence of aging. Therefore, the effect of Rg1 antioxidation on the differentiation propensity of naturally aging BMMSCs is a question that deserves additional exploration.

To test the mechanism of Rg1 on Nrf2, we chose brusatol as an inhibitor [12]. Experiments on multiple cancer cell lines [13] showed that brusatol can cooperate with multiple anti-cancer drugs to inhibit Nrf2 activity regardless of the presence of the Keap1 gene. Some studies [14, 15] have also shown that brusatol reduces Nrf2 mRNA and protein levels, while intracellular Keap1 levels remain unchanged. These findings suggest that brusatol increases the degradation of the Nrf2 protein via the ubiquitination pathway, thereby making it our preferred inhibitor.

In conclusion, the results of this experiment showed that Rg1 can modulate age-related osteogenic differentiation of BMMSCs by reducing oxidative stress damage, which is related to Nrf2-mediated antioxidative response. Specifically, in elderly people, Rg1 markedly increased osteogenic differentiation of BMMSCs by decreasing oxidative stress, while inhibiting adipogenic differentiation, which in turn may be used as a therapeutic agent to treat senile osteoporosis by improving the osteogenic differentiation of aged BMMSCs.

## 2. Materials and Methods

### 2.1. Sorting and Culture of Mesenchymal Stem Cells

The bone marrow samples were taken from the First Affiliated Hospital of Chongqing Medical University. The patients included in this study were assigned to two age groups: the young group, which consisted of individuals aged between 18 and 30 years of age, and the elderly group, which comprised individuals over 60 years of age. The enrolled patients were healthy donors undergoing one marrow aspiration or bone marrow biopsy for reasons other than blood system diseases. Patients were included in the study when their bone marrow exam showed no blood system diseases. Patients were informed about the upcoming sample collection, and informed consent was obtained from all the participants. The gender and number of participants included in the young and elderly groups were evenly distributed. The study was approved by the Ethics Committee of Chongqing Medical University (Ethics Committee Number: Chongqing, 2020-20). The collected patient information is listed in Supplementary Table 1.

The collected bone marrow was stored in EDTA anticoagulant tubes, and the mononuclear cells would be separated by density gradient centrifugation as soon as possible. The isolated cells were cultured in DMEM low-sugar medium (Gibco, USA) containing 10% fetal bovine serum (Excell, China) and 1% penicillin (Beyotime, China). Three consecutive multiplications were used to purify BMMSCs. The cells obtained after three passages are human mesenchymal stem cells. The cells obtained from three passages were identified by flowing cytometry. The method of flow cytometry is described in the previous article [16].

### 2.2. Osteogenic Differentiation and Mineralization Assay

The identified and purified BMMSCs were selected for osteogenic differentiation induction. When the cell adherence rate reached 70%, the conventional medium was replaced with osteogenesis differentiation medium (ODM) which was purchased from Cyagen Biosciences (Guangzhou, China). Performing cell culture according to reagent instructions. 72 h induction of BMMSCs for RT-PCR analysis, 14 d induction for alkaline phosphatase (ALP) staining, and 21 d induction for alizarin red staining. Rg1 was added at the beginning of induction, and the treatment time was consistent with the induction time. Cells used for ALP-staining were washed thrice prior with PBS and fixed with 4% paraformaldehyde (Sigma-Aldrich). ALP detection kit (Beyotime, China) was used for detection, according to the instructions. Cell imaging using a light microscope (Olympus Corporation, Japan).

Alizarin red stained cells were stained with Alizarin Red after PBS washing and paraformaldehyde fixation. The orange-red staining was imaged by a light microscope (Olympus Corporation, Tokyo, Japan), which indicated the location and intensity of the calcium deposition. For quantitative analysis of alizarin red, absorbance was detected at 595 nm following the distaining with 10% cetylpyridinium chloride monohydrate (Sigma) for 20 min.

### 2.3. Adipogenic Differentiation

Purified BMMSCs were used for adipogenic differentiation. The medium would be replaced with adipogenesis induction medium (ADM) (Cyagen Biosciences) when 70% adhered. We performed cell culture according to the reagent instructions 72 h induction of BMMSCs for RT-PCR analysis, and 14 d induction for Oil red O staining. Simultaneous treatment with Rg1 from the beginning of induction. Consistent duration of treatment and induction. After induction, the cells used for staining were washed thrice with PBS and fixed with 4% paraformaldehyde. Then Oil Red O staining for 15 min. The Oil Red O staining was imaged by a light microscope (Olympus Corporation, Japan). For quantification of oil red, cells would be released into the isopropanol solution. The OD value of the solution obtained by dissolving was observed under a spectrophotometry at a wavelength of 540 nm, according to a previous protocol [17].

### 2.4. Measurement of Oxidative Stress Levels

The BMMSCs at third generation were collected and lysed in an ice bath for 30 min. Lysed cells were centrifuged to collect supernatants for testing (12G, 4°C, 30 min). The chemical colorimetric kits were purchased from Beyotime Institute of Biotechnology (Shanghai, China). Performing the analysis according to the reagent manufacturer's instructions.

### 2.5. Quantitative Real-Time Reverse Transcription-Polymerase Chain Reaction (qRT-PCR)

The purified BMMSCs were lysed with TRIzol reagent (Ambion, USA). The first-strand cDNA was created through TaqMan RT reagents (TAKARA, Japan). GADPH was used for normalizing. SYBR Green Supermix (TAKARA, Japan) on iCycler Real-Time Detection System (cfx96, Bio-Rad) detected the expression levels of mRNA. The primer pair sequences used as control were as follows in Supplementary Tables 2 and 3.

### 2.6. Western Blotting

Cells were lysed with ultrasound on ice. Separation of the extract supernatant after low temperature and high-speed centrifugation. Protein concentration was detected. (Beyotime, China). Protocol of western blot was carried out as previously reported [11]. Antibody of western blotting was anti-Nrf2 (#12556) (Cell Signalling Technology, USA); Heme oxygenase-1 (HO-1) (1 : 1,000, Abcam); anti-p-Nrf2 (1 : 1,000, Bioss), NAD (P) H: quinone oxidoreductase 1 (NQO1) (1 : 1,000, Beyotime); *β*-actin (1 : 10,000, ProteinTech). Measurement of protein expression of *β*-actin as an internal reference. Relative densitometry analysis was carried out with ImageJ software.

### 2.7. Immunofluorescence

Cell dishes were treated with 0.1% Triton X-100 (Beyotime) for 30 min after washing and fixation. Goat serum blocking for 15 min. Then cells would be incubated with anti-Nrf2 antibody (1 : 200, Proteintech) overnight at 4°C. Cy3-labeled secondary antibody (1 : 200; Proteintech) was used for fluorescence labeling. The fluorescence images were captured with a Leica camera (DP73).

### 2.8. Animal Model

**Figure 1:**
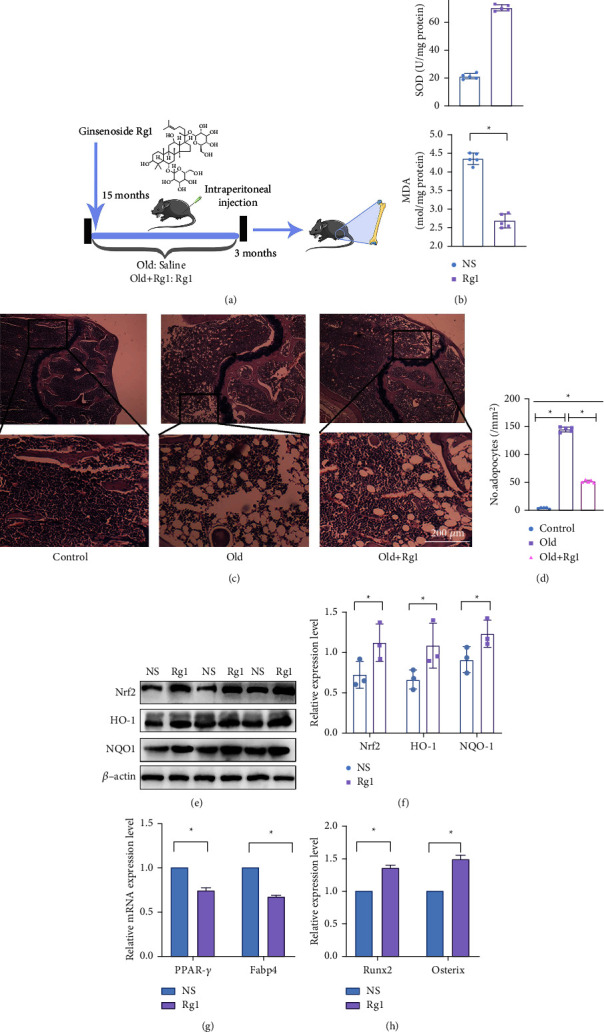
Mice treated with Rg1 reduced oxidative stress and intraosseous adipogenesis. Note: (a) A 15‐month‐old mice was used as a mice model. The treatment group was intraperitoneally injected with Rg1 at the dosage of 40 mg/kg every day for 3 months. (b) SOD and MDA levels were measured (*n* = 5). (c) Hematoxylin-Eosin staining and the number and area of adipocytes (d) in the distal femora. (e-f) Levels of Nrf2, HO-1, and NQO1 by western blot (*n* = 3). (g) Expression of Ppar-*γ* and Fabp4 by qRT‐PCR (*n* = 5). (h) Expression of Runx2 and Osx by qRT‐PCR (*n* = 5). Scale bar represent 200 *μ*m (c), Data represented by means ± SD in (b) d, (f) (g), (h). (^*∗*^*P* < 0.05, Student's *t*-test).

In vivo experiments were performed with Fifteen-month-old C57BL/6J mice (equal number of male and female mice). The experiment was divided into control and Rg1 treatment groups, with 5 mice in each group. The control group was injected intraperitoneally with saline (NS). Rg1 was injected intraperitoneally in the treatment group. The experiment lasted for 3 months and each mouse was injected once a day at a dose of 40 mg/kg of Rg1 adjusted to body weight, with the maximum amount of each injection not exceeding 200 *μ*L. The protocol of experiments. The schematic diagram of in vivo experiment is shown in (Figure 1(a)). The mice were kept in the Animal Management Centre of Chongqing Medical University. All protocols of this experiment were approved by the Animal Ethics Committee of Chongqing Medical University. The protocol of isolation and culture of BMMSCs in mice was according to a previous experiment [11].

### 2.9. Histological Assessment

10% EDTA for decalcification. The femur was then dehydrated through a gradient of 70% to 100% ethanol and then removed with xylene and embedded in paraffin. Paraffin blocks were used to make 8 *μ*m thick serial sections, after which they were stained using hematoxylin-eosin according to the instructions.

### 2.10. Statistical Analysis

Values in each histogram were presented as mean ± standard deviation (SD). Comparison between multiple groups was analyzed by one-way ANOVA. Student's *t*-test was used for comparison between two groups (GraphPad Prism 7.0; GraphPad software, La Jolla, CA, USA). Differences with a *P* value <0.05 were considered statistically significant.

## 3. Results

### 3.1. Isolation and Identification of Mesenchymal Stem Cells

**Figure 2:**
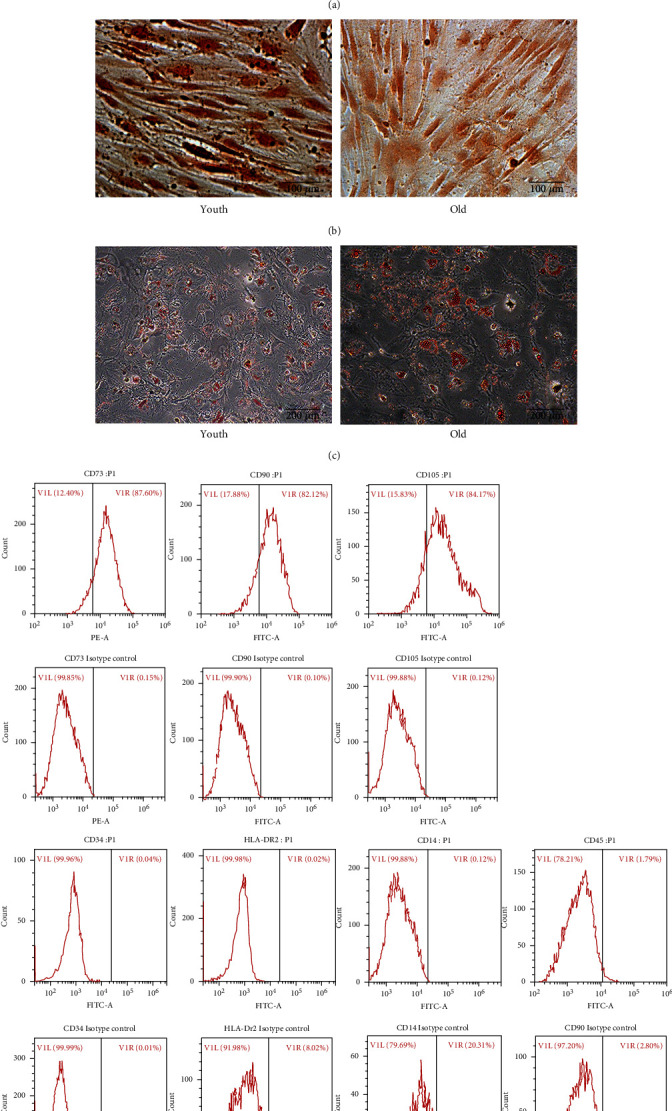
Morphology, differentiation potential, and phenotype characteristics of hBMMSCs. Note: (a) Morphology of hBMMSCs (magnification 200×). (b) ALP staining after 21 d of culture in ODM (magnification 200×). (c) Oil red O staining after 14 d of ADM induction (magnification 200×). (d) The surface markers of hBMMSCs (P3) were identified. The results indicated that the isolated cells have the common characteristics of multipotent MSCs.

A total of 60 samples of bone marrow donated by patients were collected in this experiment. After grouping by age, the genders of the subjects included in the study were evenly distributed. Each patient donated 3–5 mL bone marrow samples. In each case, 1–3 × 10^7^ mononuclear cells were obtained on average. The BMMSCs of the young group showed typical fibroblastic morphology. The shape of the BMMSCs in the elderly group was wide and flat, with prominent nuclei (Figure 2(a)).

To characterize the isolated BMMSCs, the phenotype characteristics of the third passage of BMMSCs were detected by flow cytometry. The phenotypes of BMMSCs are shown in Figure 2(d). The phenotypes of isolated cells conformed to the characteristics of mesenchymal stem cells are proposed by the International Stem Cell Association [18].

To determine the differentiation potential of the BMMSCs, these were respectively cultured in osteogenic or adipogenic for 21 d. After culturing in ODM, the BMMSCs showed abundant calcium deposits as visualized by ALP staining (Figure 2(b)). The BMMSCs cultured in adipogenesis induction medium (ADM) showed lipid vacuoles in the cytoplasm which were verified by Oil Red O staining (Figure 2(c)). These results of phenotype and differentiation potential indicated that the isolated cells have the characteristics of multipotential BMMSCs.

### 3.2. Antioxidative Ability and Osteoblast Differentiation Decrease While Adipocyte Differentiation Increases during Aging

**Figure 3:**
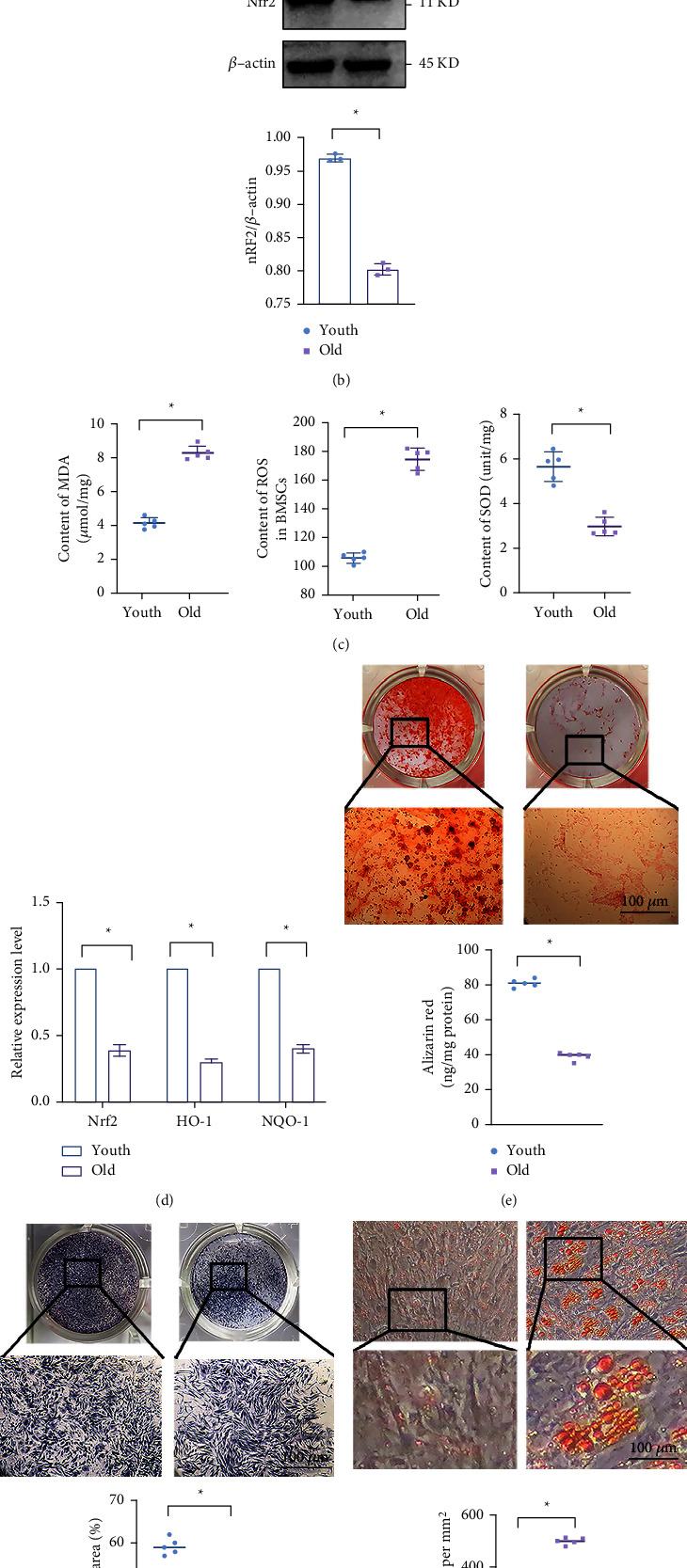
Compared to BMMSCs from young people, BMMSCs from aging people had reduced antioxidant capacity, osteogenic differentiation potential and Nrf2 expression level. Note: (a) Result of immunofluorescence showed the level of Nrf2 in the nucleus in young and elder groups. (b) Nrf2 protein expression in BMMSCs cells was measured by western blot (*n* = 3). (c) SOD enzyme levels and oxidative stress products MDA, ROS levels were compared between the elder and younger groups (*n* = 5). (d) Expression of Nrf2, HO-1, and NQO1 by qRT-PCR. (e) Alizarin red staining and quantification (*n* = 5). (f) ALP staining and quantification (*n* = 5). (g) Oil red O staining and quantification (*n* = 5). (h) Expression levels of Runx-2 and Osx by qRT-PCR (*n* = 5). (i) Expression of Fabp4 and Ppar-*γ* (*n* = 5). Scale bar represents 50 *μ*m (a), 100 *μ*m (e-g), Data represented by means ± SD in (b-i). (^*∗*^*P* < 0.05; Student's *t*-test).

Increased oxidative stress always occurs with aging. The antioxidative ability of BMMSCs in different age groups was compared. Reactive oxygen species (ROS) and malondialdehyde (MDA) levels remarkably increased in the elderly group, which indicated oxidative stress levels. Meanwhile, superoxide dismutases (SOD) activity significantly decreased, which indicated antioxidative capability (Figure 3(c)). These results implied that intracellular oxidative stress accumulated correspondingly with the aging of BMMSCs. ALP (Figure 3(f)) and alizarin red staining (Figure 3(e)) and the expression of Runt-related transcription factor 2 (Runx2) and Osteoblast transcription factor Osterix (Osx) (Figure 3(h)) both decreased in elderly groups. Oil O red staining (Figure 3(g)) and levels of peroxisome proliferator-activated receptor‐*γ* (Ppar-*γ*) and fatty acid–binding protein 4 (Fabp4) (Figure 3(i)) significantly increased, which revealed that the BMMSCs were programmed to undergo adipocyte differentiation rather than osteoblast differentiation with aging.

Specifically, the Nrf2 expression was significantly downregulated in the elder group (Figure 3(b)). Meanwhile, the Nrf2 in the nucleus was reduced with aging (Figure 3(a)). At the same time, the level of antioxidative enzymes detected by qRT-PCR showed the same trend with Nrf2 protein expression (Figure 3(d)). These results revealed that the BMMSCs in elder group has worse resistance to oxidative stress which is related to the Nrf2 pathway.

### 3.3. Rg1 Stimulates Osteoblast Differentiation and Inhibits Adipocyte Differentiation in BMMSCs

Rg1 is a natural antioxidant isolated from ginseng root which is recognized for its remarkable antioxidant capacity. To explore the appropriate concentration of Rg1 for BMMSCs, we used CCK-8 to detect the cytotoxicity of Rg1. The result indicated that at concentrations of 5, 10, and 20 *μ*g/mL, it does not affect the proliferation activity of cells (Supplementary Figure 1).

**Figure 4:**
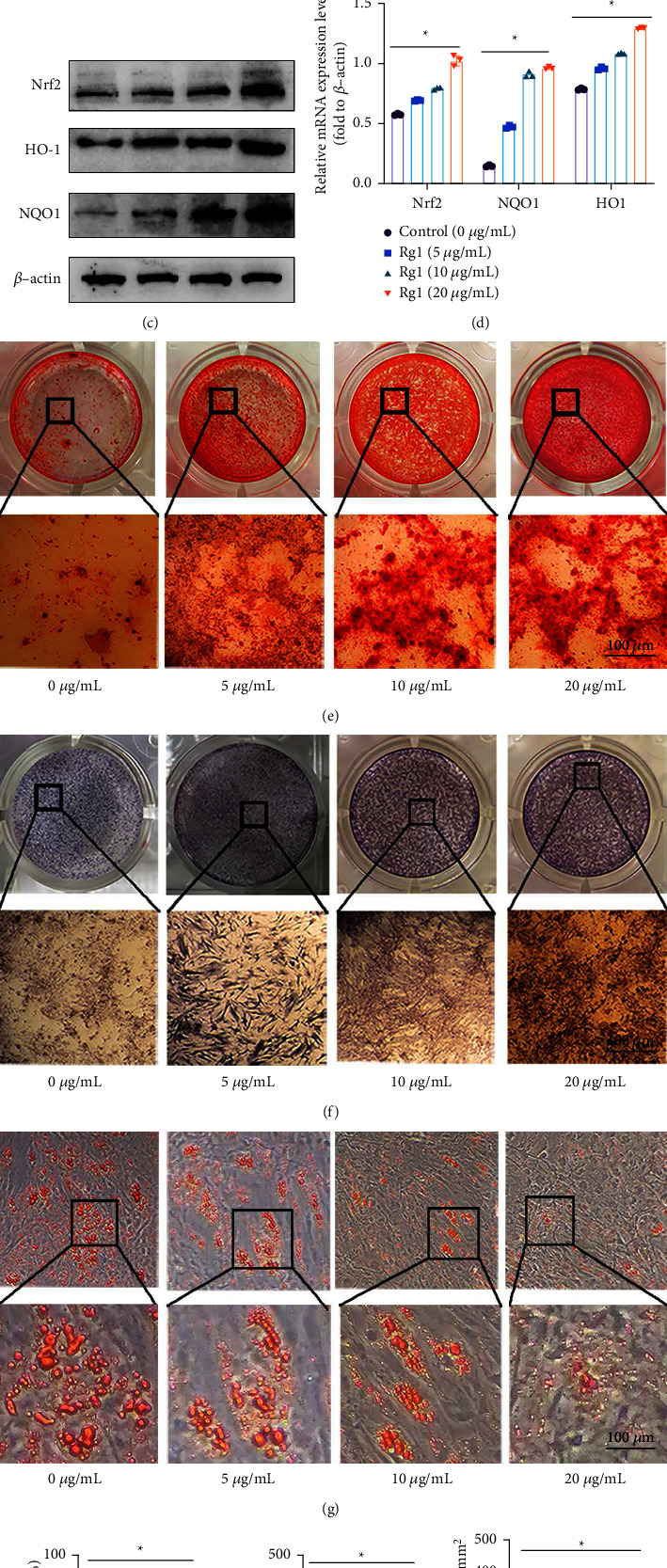
Rg1 concentration-dependently promotes osteogenic differentiation of aging BMMSCs while inhibiting adipogenic differentiation. Note: (a) Fabp4 and Ppar-*γ* level in BMMSCs with different Rg1 concentration was measured (*n* = 5). (b) Runx2 and Osx in BMMSCs with different Rg1 concentration was detected (*n* = 5). (c-d) Expression of Nrf2, NQO1, and HO-1 through western blot. *β*-Actin was internal reference (*n* = 3). (e) Alizarin red staining and (h) quantification (*n* = 5). (f) ALP staining and (i) quantification of ODM-induced BMMSCs (*n* = 5). (g) Oil red O staining and (j) quantitative analysis (*n* = 5). Scale bar represents 100 *μ*m (e), (f), (g), data represented by means ± SD in (a-d, h-j). (^*∗*^*P* < 0.05; one-way ANOVA).

ALP staining and Alizarin red staining can reflect the osteogenic differentiation ability of BMMSCs. For staining, BMMSCs would be induced by ODM with Rg1 in different concentrations. Then, the OD value and mineralized nodules were measured. The result of ALP staining (Figure 4(f)) and Alizarin red staining (Figure 4(e)) showed when compared to the 0 and 5 *μ*g/mL groups, the OD value and mineralized nodules numbers significantly increased (Figures 4(h) and 4(i)) in the 10 and 20 *μ*g/mL Rg1 groups. Osx and Runx2 is a key gene for the osteogenic differentiation of BMMSCs. The result of qRT‐PCR analysis showed that Rg1 could concentration-dependently promotes the mRNA levels (Figure 4(a)). These results show that Rg1 has a facilitative effect on osteogenic differentiation of BMMSCs.

Oil red O. staining is a classic method to detect lipid droplets. Different concentrations of Rg1 were selected to treat ADM-induced BMMSCs. Then, oil red O staining (Figure 4(g)) and quantitative analysis of lipid droplets (Figure 4(j)) were used to measure the adipogenic differentiation of BMMSCs. At the same time, the mRNA levels of key markers of adipocyte differentiation were detected by qRT‐PCR analysis (Figure 4(b)). These experiments reveal that Rg1 could concentration-dependently attenuate the adipogenic differentiation tendency of BMMSCs.

To further verify the correlation between differentiation tendency and Nrf2 levels. The levels of Nrf2 and its downstream key antioxidant enzymes were detected by western blot (Figure 4(c)). The results showed that Rg1 could concentration-dependently increases the expression level of related proteins (Figure 4(d)). In summary, Rg1 significantly inhibited adipogenic differentiation of BMMSCs and promoted osteogenic differentiation, which was related to the expression of Nrf2 protein.

### 3.4. Rg1 Regulates Differentiation of BMMSCs by Activating Nrf2 Signalling

**Figure 5:**
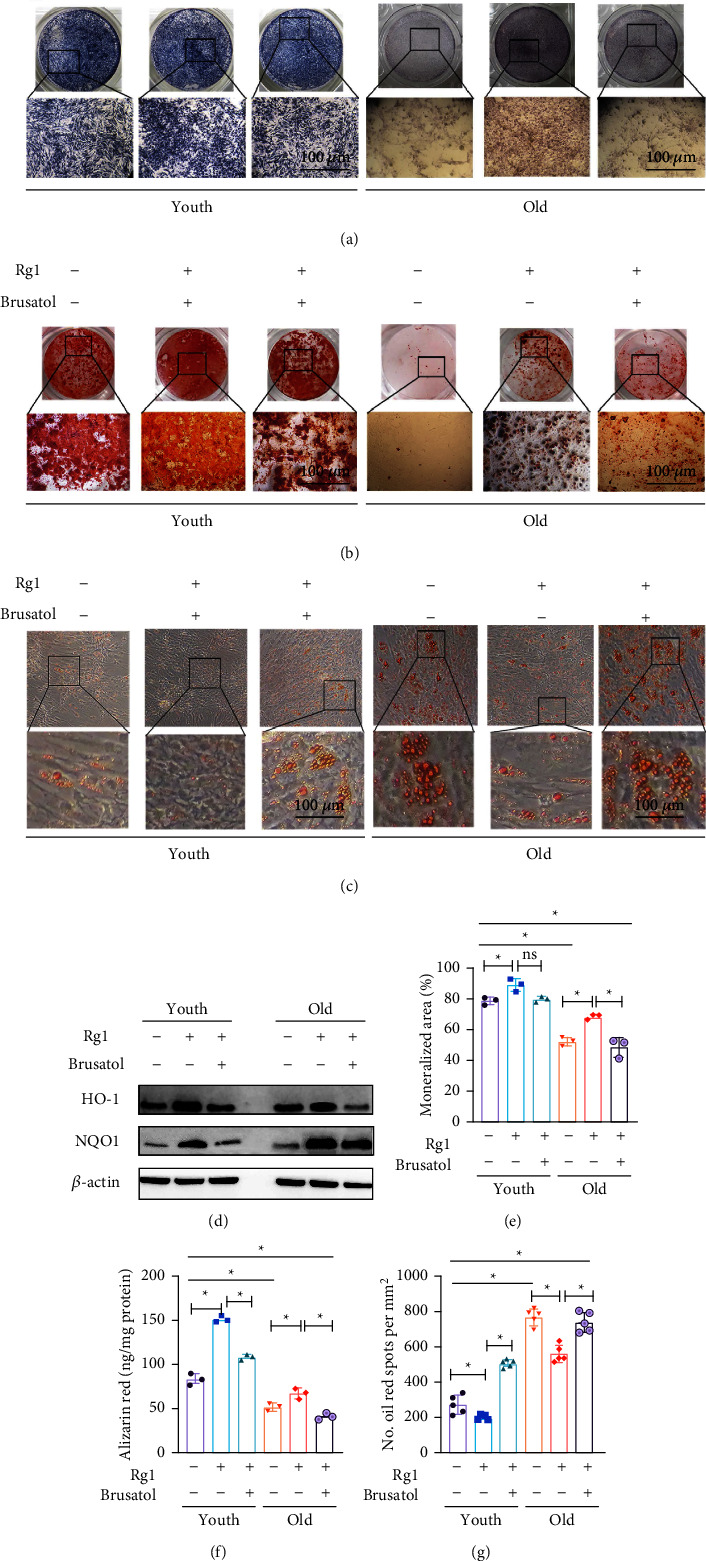
Effect of Rg1 is related to Nrf2 activation. BMMSCs treated with ODM, ODM + Rg1, or ODM + Rg1 + brusatol treatment for 18 d Note: (a) ALP staining and (d) quantitative analysis was measured (*n* = 5). (b) Alizarin red staining and (e) quantitative analysis was measured (*n* = 5). BMMSCs treated with ADM, ADM + Rg1, or ADM + Rg1 + brusatol treatment for 14 d (c) Oil red O staining and (f) quantification (*n* = 5). Scale bar represents 100 *μ*m (a-c), Data is represented by means ± SD in (d-f). (^∗^P<0.05; one-way ANOVA).

Previous reports from our research group have shown that Rg1 can reduce oxidative stress by regulating the Nrf2 pathway, thereby reducing the senescence MSCs. Multiple researches suggested that Nrf2 has a significant impact on the regulation of the balance between osteogenic and adipogenic differentiation of BMMSCs. To further explore the relationship between Nrf2 expression and Rg1 therapeutic effect, we used Nrf2 inhibitor to block Rg1 treatment. Notably, the osteogenic differentiation of BMMSCs was disrupted with the effect of Nrf2-inhibition, meanwhile, the adipogenic differentiation of BMMSCs was stimulated. Alizarin red staining (Figures 5(b) and 5(d)) and ALP staining (Figures 5(a) and 5(e)) were used to measure the osteogenic differentiation of BMMSCs. And oil red O staining (Figures 5(c) and 5(f)) was used to measure the adipogenic differentiation.

**Figure 6:**
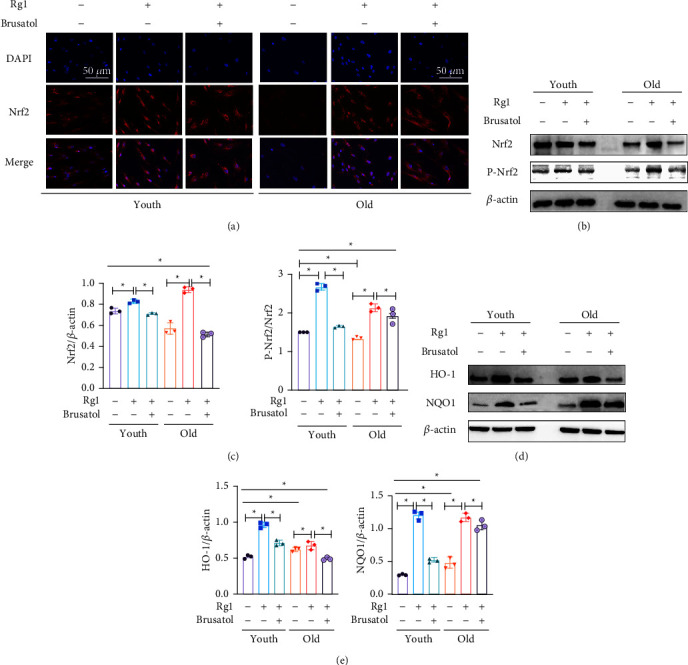
Rg1 regulates the differentiation of BMMSCs by activating Nrf2 signaling. BMMSCs were treated with ODM for 72 h. The Rg1 group was treated with 20 *μ*g/mL Rg1 for 72 h. The inhibitor group was treated with 20 *μ*g/mL Rg1 and 80 nmol/mL brusatol for 72 h. Note: (a) Immunofluorescence showing that Nrf2 expression occurs in the nucleus. (b-c) Relative expression of Nrf2 and p-Nrf2 by western blot analysis (*n* = 3), *β*-actin and Nrf2 was reference. (d-e) Relative expression levels of NQO1 and HO-1 by western blot. (*n* = 3), *β*-actin was the reference. Scale bar represents 50 *μ*m (a), Data represented by means ± SD in (d-e). (^*∗*^*P* < 0.05; one-way ANOVA).

To further observe the relationship between Rg1 and Nrf2, immunofluorescence and western blot were used to measure the nuclear translocation and phosphorylation levels of Nrf2. Immunofluorescence was used to show that the Nrf2 expression occurs in the nucleus (Figure 6(a)). When BMMSCs were treated with Rg1, the level of Nrf2 in the nucleus increased. The effect was inhibited by brusatol. The relative expression levels of Nrf2, p-Nrf2 (Figures 6(b) and 6(c)), HO-1, and NQO1 (Figures 6(d) and 6(e)) was detected with western blotting. The results show that Rg1 significantly increased their expression levels, but decreased when the inhibitor was combined. Taken together, these experiments revealed a correlation between Nrf2 signaling and the role of Rg1 in attenuating adipogenic differentiation and promoting osteogenic differentiation of BMMSCs.

### 3.5. Rg1 Treatment Reduces the Percentage of Adipocytes and Nrf2 Accumulation in Aged Mice

A 15-month-old mice was used for *in vivo* experiments. Mice were divided into two groups, the control group was treated with normal saline (NS), while the treatment group was treated with Rg1 in the concentration of 40 mg/kg. The treatment is intraperitoneal injection once a day, 200 *μ*L each time. Both groups were injected for 3 months (Figure 1(a)). After the treatment of Rg1, the oxidative stress damage significantly decreased (Figure 1(b)). Meanwhile, western blot analysis has shown that expression levels of Nrf2, HO-1, and NQO1 in the aging + Rg1 group were significantly higher than aging group (Figures 1(e) and 1(f)). The results of the qRT‐PCR analysis revealed that the osteogenesis-related genes increased in the aging + Rg1group, while the adipogenic-related genes decreased (Figures 1(g) and 1(h)). HE staining showed that the bone marrow of aging group had undergone significant fortification. In addition, Rg1 treatment decrease the level of fortification in the bone marrow (Figures 1(c) and 1(d)). The experiments showed that long-term Rg1 treatment can alleviate oxidative stress damage in aging mice, thereby improving age-related bone loss.

## 4. Discussion

Aging is a naturally occurring process. In this process, cell damage in various organs is accompanied. Stem cells also undergo aging that limits their proliferation and differentiation capability, as well as responsiveness to external signals. The decline in function of stem cells is an important driver of age-related pathologies. As an important organ of the human body, bones mainly show bone loss during aging. Oxidative stress is an important cause of dysregulation of aging-related bone homeostasis [19–21] that destroys the lineage commitment of BMMSCs and reduces osteoblast differentiation [1, 22]. To conclude, this study demonstrates that the antioxidant effect of Rg1 can alleviate oxidative stress in BMMSCs, thereby ameliorating age-related osteogenic differentiation disorders.

Previous studies have shown that oxidative stress damage is an important factor affecting the balance of bone marrow osteogenesis and adipogenicity [23]. We isolated BMMSCs from young and elderly groups and compared with young people, BMMSCs in the elderly group had lower levels of resistance to oxidative stress enzymes; at the same time, the group also showed a decrease in osteogenic differentiation potential. Some researches also indicate that Nrf2 has a strong correlation to aging-related diseases as the Nrf2 antioxidant pathway was the main mechanism of the aging-related disorder. Impairment of Nrf2 expression level may be associated with oxidative stress and its associated premature cellular aging, including bone loss [24].

Nrf2 is an important pathway for regulating oxidative stress levels [25]. Activated Nrf2 forms bind to an antioxidant response element (ARE), which regulates the expression of various antioxidative stress and chemical damage-related enzymes, such as NQO1, and HO-1 subunit [26, 27], and regulates the expression of these genes in various organs, including bone [28, 29]. Researches have shown that the activation of Nrf2 can shift the switch between osteogenic lineage and adipogenic lineage in MSCs [25, 28]. In addition, animal experiments also show that loss of Nrf2 would lead to bone loss [30, 31]. In this study, we isolated BMMSCs from different ages to assess the correlation among Nrf2, oxidative stress, and age-related cell fate. The Nrf2 levels in the elderly group were lower and accompanied by increasing oxidative stress and enhanced adipogenic differentiation, which suggests the important role of Nrf2 in BMMSC lineage commitment.

Osteoporosis is a disease that puts patients at increased risk of fractures, which greatly increases mortality and health-care costs in the elderly [32]. Aging of BMMSCs is an important target for the treatment of senile osteoporosis. In theory, targeted therapy for aging MSCs can help relieve senile osteoporosis. However, to date, osteoporosis treatment drugs targeting aging cells have rarely been used. Ginseng is a widely used traditional Chinese medicine. Among a variety of ginsenosides, Rg1 is proven to be an important pharmacologically active ingredient that has been known for its antioxidant activity. Previous researches have revealed that Rg1 imparts a protective effect on the osteogenic lineage [7]. Some researches have shown that Rg1 can effect as an regulator of mesenchymal stem cells [33]. In our previous studies, we found that Rg1 can improve the senescence of hematopoietic stem/progenitor cells by improving oxidative stress damage [34]. At the same time, studies have shown that mesenchymal stem cells participate in the construction of the hematopoietic microenvironment [35]. The differentiation direction of mesenchymal stem cells affects the changes in hematopoietic microenvironment and the function of hematopoietic stem/progenitor cells. This study analyzed the effect of Rg1 antioxidative stress on the differentiation and cell fate of mesenchymal stem cells and provided clues of reference significance for elucidating the specific mechanism of ginsenoside Rg1 for promoting hematopoietic function.

In this study, we found that BMMSCs from the elderly people had higher oxidative stress than the younger, which was accompanied by the imbalance of osteogenic and adipogenic in BMMSCs. Rg1 could reverse this effect in a dose-dependent manner. However, when treating with an Nrf2 inhibitor, the effect of Rg1 treatment was not observed.

In conclusion, Rg1 reduces the oxidative stress damage in BMMSCs by regulating the Nrf2 signaling pathway and alleviating the dysfunction of MSCs caused by aging. In addition, we verified the antiosteoporosis activity of Rg1 by observing the recovery of bone loss in Rg1-treated mice, which was closely related to restoring the antioxidant capacity of bone.

## 5. Conclusions

**Figure 7:**
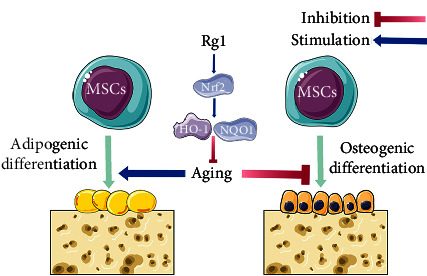
Rg1 promotes adipogenic differentiation and inhibits lipogenic differentiation in aging BMMSCs by attenuating oxidative stress damage. This effect is associated with the activation of the Nrf2 signaling and its downstream antioxidant enzymes.

In summary, this study established the medicinal value of Rg1 in reducing oxidative stress to improve osteogenic differentiation of aging hBMMSCs that involves the Nrf2 signaling pathway (Figure 7). These findings could provide a new treatment for age-related osteoporosis.

## Data Availability

The data used to support this study can be obtained from the corresponding author upon request.

## Supplementary Materials

Supplementary Materials Supplementary Table 1 Information of Patients. Supplementary Table 2 Primers of human for quantitative RT-PCR. Supplementary Table 3 Primers of mice for quantitative RT-PCR. Supplementary Figure 1. A The chemical structure of ginsenoside Rg1. B Rg1 drug concentration was detected by CCK-8. Data are presented as mean ± SD (*n* = 3/group). Statistical significance was determined using ANOVA, ^*∗*^*P* < 0.05.
