# Supplementary material for: Ginsenoside Rg1 Reduces Oxidative Stress Via Nrf2 Activation to Regulate Age-Related Mesenchymal Stem Cells Fate Switch Between Osteoblasts and Adipocytes
Source: Evid Based Complement Alternat Med. 2022 Oct 11;2022:1411354. doi: 10.1155/2022/1411354 (PMC9578818; doi:10.1155/2022/1411354)
Supplement: Supplementary Materials — Supplementary Table 1 Information of Patients. Supplementary Table 2 Primers of human for quantitative RT-PCR. Supplementary Table 3 Primers of mice for quantitative RT-PCR. Supplementary Figure 1. A The chemical structure of ginsenoside Rg1. B Rg1 drug concentration was detected by CCK-8. Data are presented as mean ± SD (n = 3/group). Statistical significance was determined using ANOVA, ∗P < 0.05. [file 1411354.f1.zip › Supplementary Tables1-3.docx]

Supplementary Table 1 Information of Patients

| No. | Name | Age | Reason | Gender | Diagnostic result |
| --- | --- | --- | --- | --- | --- |
| 1 | Mei* Liu | 61 | anemia | Female | （—） |
| 2 | Yu* Gao | 65 | anemia | Male | （—） |
| 3 | Rong* Yuan | 66 | anemia | Female | （—） |
| 4 | * Hu | 28 | anemia | Female | （—） |
| 5 | Wu* Liu | 65 | anemia | Male | （—） |
| 6 | Li* Tang | 18 | anemia | Female | （—） |
| 7 | Tian* Hou | 79 | anemia | Male | （—） |
| 8 | Jia* Han | 20 | anemia | Female | （—） |
| 9 | Zai* Peng | 66 | anemia | Female | （—） |
| 10 | Ze* Du | 65 | anemia | Male | （—） |
| 11 | Yong* Dong | 66 | fever | Female | （—） |
| 12 | Yi* Huang | 21 | anemia | Female | （—） |
| 13 | Ming* Zhang | 24 | anemia | Female | （—） |
| 14 | Chang* Qiu | 61 | anemia | Female | （—） |
| 15 | Feng* Li | 27 | anemia | Female | （—） |
| 16 | Shao* Feng | 84 | anemia | Male | （—） |
| 17 | Ming* Xiao | 23 | anemia | Male | （—） |
| 18 | Dai* Zhang | 68 | anemia | Female | （—） |
| 19 | Su*Li | 70 | anemia | Male | （—） |
| 20 | Chun* Wu | 26 | anemia | Female | （—） |
| 21 | Kai* Wu | 84 | anemia | Male | （—） |
| 22 | *Xie | 26 | anemia | Male | （—） |
| 23 | Xin* Wang | 67 | anemia | Female | （—） |
| 24 | Gan* Liu | 20 | anemia | Male | （—） |
| 25 | Xing* Tang | 84 | anemia | Female | （—） |
| 26 | *Zheng | 84 | fever | Male | （—） |
| 27 | *Zheng | 26 | anemia | Female | （—） |
| 28 | Guo* He | 64 | anemia | Male | （—） |
| 29 | Su* Lang | 72 | anemia | Female | （—） |
| 30 | *Yang | 26 | anemia | Female | （—） |
| 31 | Zhong* Huang | 70 | anemia | Male | （—） |
| 32 | Yao* Pu | 62 | anemia | Male | （—） |
| 33 | Xiao* Tang | 27 | anemia | Male | （—） |
| 34 | *Qing | 21 | anemia | Male | （—） |
| 35 | Zhi* Xu | 73 | anemia | Female | （—） |
| 36 | *Yang | 25 | anemia | Male | （—） |
| 37 | Neng* Tian | 29 | anemia | Female | （—） |
| 38 | Lin* Ma | 27 | anemia | Female | （—） |
| 39 | *Chen | 23 | anemia | Male | （—） |
| 40 | Li*Xiang | 62 | anemia | Female | （—） |
| 41 | Rong* Sun | 66 | anemia | Male | （—） |
| 42 | *Wang | 24 | anemia | Female | （—） |
| 43 | Ming* Yang | 62 | fever | Female | （—） |
| 44 | *Li | 24 | anemia | Male | （—） |
| 45 | Hong* Zhang | 68 | anemia | Male | （—） |
| 46 | Gu* Yu | 24 | anemia | Male | （—） |
| 47 | *Gong | 72 | anemia | Female | （—） |
| 48 | *Yuan | 21 | anemia | Female | （—） |
| 49 | Xiao* Tian | 24 | anemia | Male | （—） |
| 50 | Ming* Yang | 61 | anemia | Male | （—） |
| 51 | Wei* Yang | 63 | anemia | Female | （—） |
| 52 | Hong* Ye | 21 | anemia | Male | （—） |
| 53 | Wei* Dai | 23 | anemia | Male | （—） |
| 54 | *Liu | 25 | anemia | Female | （—） |
| 55 | Ji* Zhang | 24 | anemia | Male | （—） |
| 56 | Yan* Hou | 23 | anemia | Female | （—） |
| 57 | Zai* Yang | 24 | anemia | Male | （—） |
| 58 | Yong* Zheng | 66 | anemia | Male | （—） |
| 59 | Xiao* Chen | 25 | anemia | Male | （—） |
| 60 | Shu* Zhang | 79 | anemia | Female | （—） |

Supplementary Table 2 Primers of human for quantitative RT-PCR

| Gene | \| Primer \|  \| \| --- \| --- \| | Primer (5’–3’) |
| --- | --- | --- | --- | --- |
| RUNX2 | Forward | 5’-AAGTGCGGTGCAAACTTTCT-3’ |
|  | Reverse | 5’-TCTCGGTGGCTGCTAGTGA-3’ |
| Osterix | Forward | 5’-GCCAGAAGCTGTGAAACCTC-3’ |
|  | Reverse | 5’-GCTGCAAGCTCTCCATAACC-3’ |
| PPAR-γ | Forward | 5’-AACTTACAAACGCAAGGAACTCT-3’ |
|  | Reverse | 5’-TCAGAGTTCCTTGCGTTTGTAAGT-3’ |
| Fabp4 | Forward | 5’- AATGAAAAATGCTGCTTTCTATAGA-3’ |
|  | Reverse | 5’- TCTATAGAAAGCAGCATTTTTCATT-3’ |
| NRF2 | Forward | 5’-TCAGCGACGGAAAGAGTATGA-3’ |
|  | Reverse | 5’-CCACTGGTTTCTGACTGGATGT-3’ |
| NQO1 | Forward | 5’-GAAGAGCACTGATCGTACTGGC-3’ |
|  | Reverse | 5’-GGATACTGAAAGTTCGCAGGG-3’ |
| HO-1 | Forward | 5’-ATGCCCCAGGATTTGTCAGA-3’ |
|  | Reverse | 5’-AAGTAGACAGGGGCGAAGAC-3’ |
| GADPH | Forward | 5’-TCCTAGCACCATGAAGATC-3’ |
|  | Reverse | 5’-AAACGCAGCTCAGTAACAG-3’ |

Supplementary Table 3 Primers of mice for quantitative RT-PCR

| Gene | | \| Primer \|  \| \| --- \| --- \| | | Primers |
| --- | --- | --- | --- | --- | --- | --- |
| Actin | | Forward | | 5’-GGCTGTATTCCCCTCCATCG-3’ |
|  | | Reverse | | 5’-CCAGTTGGTAACAATGCCATGT-3’ |
| PPAR-γ | | Forward | | 5’-ATGGTT GACACAGAGATGC-3’ |
|  | | Reverse | | 5’-GAATGCGAGTGGTCTTCC-3’ |
| Fabp4 | | Forward | | 5’-AAGGTGAAGAGCATCATAACCCT-3’ |
|  | | Reverse | | 5’-TCACGCCTTTCATAACACATTCC-3’ |
| RUNX2 | | Forward | | 5’-CACTGAAACCTCTAAGATCCTTTGA-3’ |
|  | | Reverse | | 5’-TCAAAGGATCTTAGAGGTTTCAGTG-3’ |
| Osterix | | Forward | | 5’-AGCCTCAAATTGTCCCTATTCTTGG-3’ |
|  | | Reverse | 5’-CCAAGAATAGGGACAATTTGAGGCT-3’ |  |
| Nrf2 | | Forward | 5’-TCCGCTGCCATCAGTCAGTC-3’ |  |
|  | | Reverse | 5’-ATTGTGCCTTCAGCGTGCTTC-3’ |  |
| NQO1 | | Forward | 5’-CAAGTTTGGCCTCTCTGTGG-3’ |  |
|  | | Reverse | 5’-AAGCTGCGTCTAACTATATGT-3’ |  |
| HO-1 | | Forward | 5’-AACAAGCAGAACCCAGTCTATGC-3’ |  |
|  | | Reverse | 5’-AGGTAGCGGGTATATGCGTGGGCC-3’ |  |
