# Supplementary figures and images for: Ginsenoside Rg1 Reduces Oxidative Stress Via Nrf2 Activation to Regulate Age-Related Mesenchymal Stem Cells Fate Switch Between Osteoblasts and Adipocytes
Source: Evid Based Complement Alternat Med. 2022 Oct 11;2022:1411354. doi: 10.1155/2022/1411354 (PMC9578818; doi:10.1155/2022/1411354)

**A**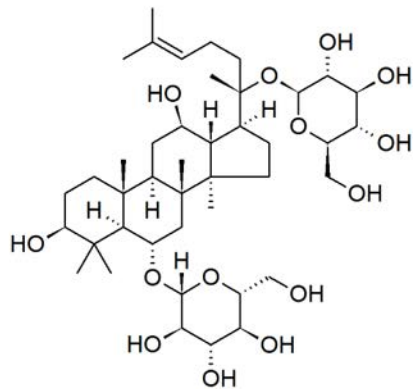**B**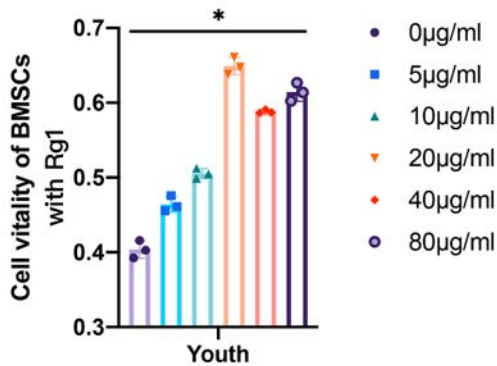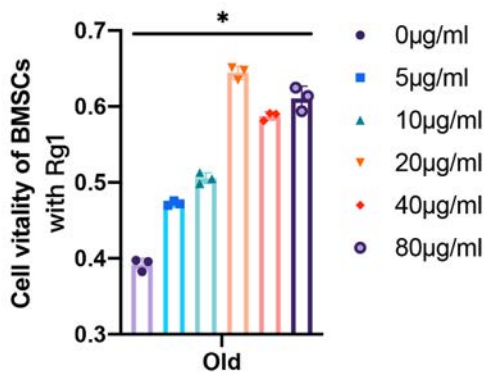

Supplement: Supplementary Materials — Supplementary Table 1 Information of Patients. Supplementary Table 2 Primers of human for quantitative RT-PCR. Supplementary Table 3 Primers of mice for quantitative RT-PCR. Supplementary Figure 1. A The chemical structure of ginsenoside Rg1. B Rg1 drug concentration was detected by CCK-8. Data are presented as mean ± SD (n = 3/group). Statistical significance was determined using ANOVA, ∗P < 0.05. [file 1411354.f1.zip › Supplementary Figure 1.pdf]
